# The Role of Endothelial Activation and Stress Index (EASIX) for Predicting Contrast-Induced Nephropathy and In-Hospital Mortality in Patients with ST-Segment Elevation Myocardial Infarction Undergoing Primary Percutaneous Coronary Intervention

**DOI:** 10.3390/diagnostics16081123

**Published:** 2026-04-09

**Authors:** Kurtulus Karauzum, Veysel Ozan Tanık, Alperen Tas, Didar Mirzamidinov, Uygur Simsek, Ebrar Gencer, Furkan Celik, Naila Badalova, Fatih Cihat Buyukbas, Irem Yilmaz, Goksel Kahraman, Tayfun Sahin, Ertan Ural

**Affiliations:** 1 Cardiology Department, Kocaeli University Hospital, 41200 İzmit, Türkiye; dmirzamidinov@gmail.com (D.M.); uygursmsk@gmail.com (U.S.); ebrargencer1309@gmail.com (E.G.); furkancelik.gs@gmail.com (F.C.); karauzumirem@gmail.com (I.Y.); gokselkahraman@hotmail.com (G.K.); tayfunsa@yahoo.com (T.S.); ertanural@yahoo.com (E.U.); 2 Cardiology Department, Etlik City Hospital, 06170 Ankara, Türkiye; drozantanik@gmail.com (V.O.T.); alperentas555@hotmail.com (A.T.); nailabadalova71@gmail.com (N.B.); fcihat08@gmail.com (F.C.B.)

**Keywords:** primary percutaneous coronary intervention, inflammation, contrast-induced nephropathy

## Abstract

**Background**: The endothelial activation and stress index (EASIX), derived from the serum lactate dehydrogenase, creatinine, and platelet counts, is a composite biomarker for endothelial dysfunction and systemic stress. It has been developed to predict clinical outcomes in hematologic malignancies. This study aimed to investigate the EASIX’s predictive role in contrast-induced nephropathy (CIN) and in-hospital mortality in patients with ST-segment elevation myocardial infarction (STEMI) undergoing primary percutaneous coronary intervention (PCI). **Methods**: A total of 1552 patients with STEMI who underwent primary PCI were retrospectively included. The patients were divided into two groups: CIN (+) and CIN (−). Baseline demographic, laboratory, clinic, and procedural variables were compared between the two groups. Logistic regression analysis was performed to identify independent predictors of CIN and in-hospital mortality, while receiver operating characteristic (ROC) curves were used to determine the optimal EASIX cut-off values. **Results**: CIN developed in 7.6% (*n* = 118) of the study population, and these patients had significantly increased EASIX scores. Those with CIN were older and exhibited higher rates of diabetes mellitus, chronic kidney disease (CKD), and decreased left ventricular ejection fraction (LVEF) (all *p* < 0.001). In multivariable analysis, age (OR 1.053), CKD (OR 1.338), reduced LVEF (OR 0.965), and EASIX (OR 2.467) independently predicted CIN. EASIX > 0.93 demonstrated strong discriminatory ability (AUC 0.785; sensitivity 72% and specificity 72%). EASIX also independently predicted in-hospital mortality (OR 3.592), with an optimal cut-off > 0.88 (AUC 0.774). **Conclusions**: By integrating markers of renal function, endothelial activation, and systemic stress, EASIX may serve as a useful and reliable indicator for predicting CIN development and in-hospital mortality in STEMI patients undergoing primary PCI.

## 1. Introduction

Contrast-induced nephropathy (CIN) is one of the most important and serious complications of invasive angiographic procedures [1,2]. CIN refers to acute kidney injury (AKI) developing subsequent to exposure to contrast media without other identifiable causes [1,2]. Reported CIN incidence in the overall population is between 1% and 6% but may exceed 50% in high-risk groups, including patients with chronic kidney disease (CKD), older age, diabetes mellitus, hemodynamic instability, or heart failure (HF) [3,4,5]. CIN has been linked to prolonged hospitalization, increased need for dialysis, acute myocardial infarction (AMI), recurrent revascularization, and mortality [6,7]. The mechanisms of CIN have not been fully clarified, but growing evidence suggests that inflammation and endothelial dysfunction are pivotal in its development [8,9]. Contrast exposure induces oxidative stress, endothelial activation, and tubular epithelial injury, all of which trigger local and systemic inflammatory responses [8,9]. Based on these, several inflammation-related biomarkers, such as red cell distribution width, neutrophil-to-lymphocyte ratio (NLR), C-reactive protein (CRP), and systemic immune-inflammation index (SII), have been described to be associated with the CIN development in patients undergoing percutaneous coronary intervention (PCI) [10,11,12,13]. Early and accurate prediction of its occurrence is critical to improve clinical outcomes for the application of preventive strategies prior to PCI. The identification of useful and reliable indicators is therefore essential, as they allow clinicians to recognize high-risk patients requiring more intensive monitoring, tailored hydration protocols, and contrast-sparing techniques. The endothelial activation and stress index (EASIX), computed from serum lactate dehydrogenase (LDH), serum creatinine, and platelet count, was recently proposed as a surrogate marker of endothelial dysfunction and systemic stress [14]. Earlier studies have demonstrated the prognostic role of EASIX in various clinical settings, including hematologic malignancies, systemic inflammatory and cardiovascular diseases [14,15,16,17,18,19,20]. However, its role in predicting CIN and short-term outcomes in patients with ST-segment elevation myocardial infarction (STEMI) undergoing primary PCI remains underexplored. This study aimed to evaluate the predictive value of the EASIX for CIN and in-hospital mortality in STEMI patients who underwent primary PCI.

## 2. Materials and Methods

### 2.1. Study Population

A total of 1819 consecutive patients presenting with STEMI and treated with primary PCI in the cardiology department between March 2020 and May 2025 were retrospectively included in the study. Patients were excluded if they had received thrombolytic therapy before PCI, were chronically dependent on renal replacement therapy, had active or chronic infection, hepatobiliary disease, hematologic disorders, malignancy, chronic inflammatory conditions, regular use of steroids or nonsteroidal anti-inflammatory drugs, a history of organ transplantation, or lacked creatinine measurements within the first 72 h after primary PCI. Following the exclusion of 267 patients, the remaining 1552 individuals constituted the final study cohort.

### 2.2. Study Protocol

Demographic data and clinical history were collected, including sex, age, body mass index, smoking status, diabetes mellitus, hypertension, chronic obstructive pulmonary disease (COPD), CKD, chronic HF, medications on admission, and any prior PCI. The STEMI diagnosis was established at presentation following guideline criteria, including either new left bundle branch block or ≥1 mm ST-segment elevation in at least two adjacent ECG leads, along with clinical signs of myocardial ischemia and/or raised cardiac necrosis markers [21]. Before the procedure, every patient received a 300 mg loading dose in combination with a P2Y12 receptor inhibitor, which was either clopidogrel (600 mg), prasugrel (60 mg), or ticagrelor (180 mg). Coronary angiography used femoral or radial access at the operator’s discretion, and primary PCI was conducted via standard techniques in line with international guidelines. Intravenous unfractionated heparin (70–100 U/kg) was administered throughout the procedure to reach an activated clotting time of 250–300 s, or 200–250 s in cases involving tirofiban administration. The administration of tirofiban was determined at the operator’s discretion. All procedures utilized a nonionic, low-osmolar contrast agent (iohexol, Omnipol 300 mg I/mL; Polifarma, Istanbul, Türkiye). All patients received evidence-based adjunctive pharmacotherapy in accordance with current guideline recommendations, unless contraindicated. Post-procedurally, patients received intravenous hydration with 0.9% sodium chloride at 1 mL/kg/h for 12 h, or 0.5 mL/kg/h if left ventricular ejection fraction (LVEF) < 40%. Patients presenting cardiogenic shock and/or acute HF did not receive hydration [22].

Venous blood samples required for laboratory analyses were obtained prior to the primary PCI procedure. Complete blood counts were analyzed in samples collected in EDTA-containing tubes within 30 min via an automated hematology analyzer (Sysmex K-1000, Kobe, Japan). Conventional laboratory analyses (Roche Diagnostic Modular Systems, Tokyo, Japan) were used to assess biochemical parameters such as serum creatinine, albumin, lipid profile, and glucose. The EASIX value was computed with the formula: serum lactate dehydrogenase [U/L] × creatinine [mg/dL]/platelet count [10^9^/L] [14].

All routine laboratory parameters except peak troponin were examined on hospital admission before the procedure. Peak troponin I values were assessed 18–24 h following the procedure. Estimated glomerular filtration rate (eGFR) was determined via the Modification of Diet in Renal Disease equation [23]. Transthoracic echocardiography was conducted during the initial 24 h post-procedure, and LVEF was evaluated via the modified Simpson’s method as recommended in current guidelines [24].

CIN was defined as a rise in serum creatinine by ≥0.5 mg/dL or ≥25% from baseline during the initial 72 h following contrast administration [25]. Using these definition criteria, the cohort was stratified into two groups: CIN (+) and CIN (−). Hospital records and patient files provided all clinical, laboratory, and procedural data.

### 2.3. Statistical Analysis

Data were analyzed employing SPSS version 22.0 (IBM Corp., Armonk, NY, USA). Assessment of normality for continuous variables was performed via the Kolmogorov–Smirnov test, with normally distributed data summarized as mean ± SD and skewed variables as median (min–max) for skewed data, and categorical variables as frequencies and percentages. Continuous variables in CIN and non-CIN groups were evaluated via Student’s *t*-test or Mann–Whitney U test, and categorical variables were examined via chi-square or Fisher’s exact test. Baseline characteristics across EASIX quartiles (≤25th, 25th–75th, ≥75th percentiles) were compared via one-way ANOVA or Kruskal–Wallis tests with suitable post hoc analyses for continuous data and chi-square tests for categorical data. Univariable logistic regression analyses were performed to identify variables associated with CIN and in-hospital mortality. Variables with *p* < 0.05 in univariable analyses, as well as those considered clinically relevant, were entered into the multivariable logistic regression models. Accordingly, the following variables were included in the multivariable model for CIN: age, diabetes mellitus, left ventricular ejection fraction, chronic renal failure, LDL cholesterol, failed PCI, and EASIX. For the in-hospital mortality analysis, the initial multivariable model included age, diabetes mellitus, left ventricular ejection fraction, failed PCI, CIN, and EASIX. A backward stepwise (Wald) elimination procedure was applied, with variables sequentially removed if *p* > 0.05 until all remaining predictors met the retention criterion.

To avoid multicollinearity, variables reflecting similar clinical or laboratory domains were not included simultaneously in the multivariable model. Multicollinearity was further assessed using variance inflation factor (VIF) values, and all variables in the final model had VIF values close to 1 (all < 2), indicating no significant multicollinearity.

Receiver operating characteristic (ROC) curve analyses were performed to evaluate the discriminatory ability of EASIX and other predictors for CIN and in-hospital mortality. The area under the curve (AUC) and 95% confidence intervals (CI) were calculated. Optimal cut-off values of EASIX were determined by Youden’s index, and corresponding sensitivity and specificity were reported.

Survival analysis was conducted using the Kaplan–Meier method. Patients were stratified into groups according to both the ROC-derived EASIX cut-off value and EASIX quartiles, and survival curves were generated. Differences between survival curves were assessed using the log-rank test. A two-sided *p* < 0.05 was considered statistically significant.

## 3. Results

In the present study, 1552 patients undergoing primary PCI for STEMI were included, among whom CIN developed in 118 patients (7.6%). Table 1 summarizes baseline clinical and demographic characteristics grouped by CIN status. Patients with CIN were older than those without CIN (64.85 ± 12.40 vs. 58.08 ± 11.46 years; *p* < 0.001). Gender, hypertension, COPD, smoking, and blood pressures on admission were similar between groups, while diabetes and CKD were more frequent in patients with CIN than those without CIN (39.8% vs. 21.8%; *p* < 0.001 and 8.5% vs. 1.0%; *p* < 0.001, respectively). Frequencies of GPIIbIIIa usage and prior PCI did not show a statistically significant difference between the groups (*p* = 0.594 and *p* = 0.554, respectively). Compared to patients without CIN, those with CIN exhibited significantly reduced LVEF (42.95 ± 13.30% vs. 47.85 ± 9.94%, *p* < 0.001). Use of statins, beta-blockers, and angiotensin-converting enzyme inhibitors (ACEi) or angiotensin receptor blockers (ARB) on admission was comparable between the groups. Also, there were no statistical differences in terms of culprit vessel of the STEMI and cardiogenic shock and/or acute HF between the two groups.

Table 2 summarizes the laboratory findings for the study cohort. Patients who developed CIN had significantly lower eGFR values (99.16 ± 29.35 vs. 72.36 ± 23.77; *p* < 0.001) and higher admission glucose levels [170 (90–608) vs. 133 (53–693); *p* < 0.001] compared with those without CIN. Baseline serum creatinine, hemoglobin, and lipid profile levels were comparable between groups.

Peak troponin level was slightly higher in the patients with CIN than those without CIN, but it was not statistically significant (3.91 ± 1.68 vs. 3.68 ± 1.70; *p* = 0.092). Regarding inflammatory cell counts and derived indices, patients with CIN had significantly higher platelet counts (234.78 ± 69.50 vs. 247.14 ± 81.50; *p* = 0.006), LDH (200.51 ± 34.33 vs. 256.48 ± 41.24; *p* < 0.001), and EASIX [0.74 (0.14–35.96) vs. 1.24 (0.46–5.27); *p* < 0.001] than patients without CIN.

Table 3 presents baseline characteristics stratified by quartiles, with significant differences among the two groups regarding age, gender, diabetes, eGFR, LVEF, platelet count, creatinine, and LDH levels. Patients in the >75th quartile group were of higher age, more frequently male, and had greater rates of diabetes, CKD, and COPD. They also exhibited lower LVEF and higher platelet counts, LDH, and creatinine levels compared with patients in the <25th quartile and 25–75th quartile groups.

Table 4 shows the clinical outcomes of univariable and multivariable logistic regression examining CIN. The variables age (*p* < 0.001; OR 1.052 95% CI 1.035–1.070), diabetes (*p* < 0.001; OR 2.371 95% CI 1.606–3.499), failed PCI (*p* = 0.024; OR 2.041 95% CI 1.100–3.787), LVEF (*p* < 0.001; OR 0.958 95% CI 0.940–0.976), LDL (*p* = 0.002; OR 1.008 95% CI 1.003–1.013), CKD (*p* < 0.001; OR 1.225 95% CI 1.089–1.361), and EASIX (*p* < 0.001; OR 1.771 95% CI 1.370–2.290), which are shown to have significant differences, were linked to CIN in univariate analysis. Multivariate analysis identified age (*p* < 0.001, OR 1.053, 95% CI 1.031–1.075), LVEF (*p* = 0.001, OR 0.965, 95% CI 0.944–0.986), CKD (*p* = 0.045, OR 1.338, 95% CI 1.098–1.678), and EASIX (*p* < 0.001, OR 2.467, 95% CI 1.709–3.560) as independent predictors for CIN.

**Figure 1:**
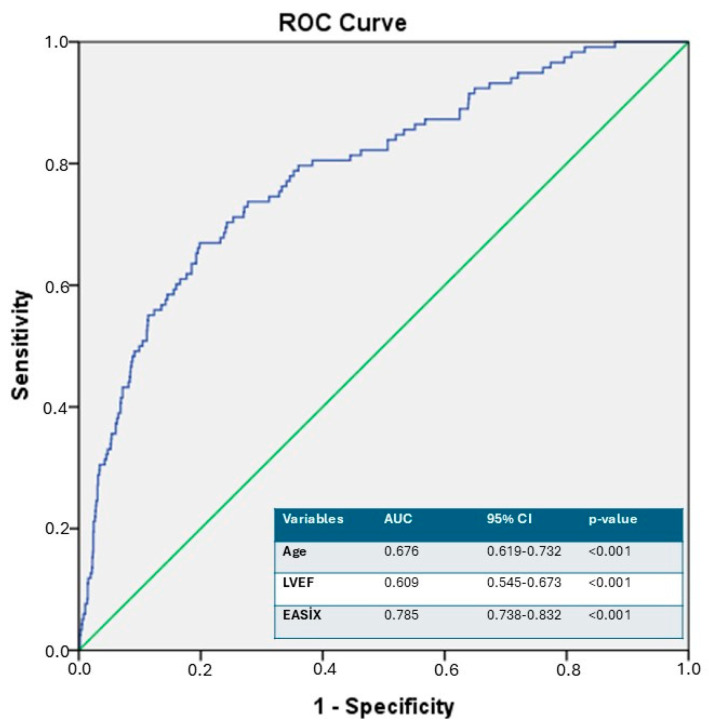
The ROC curve analysis of EASIX for predicting contrast-induced nephropathy. ROC: receiver operating characteristic, EASIX: endothelial activation and stress index, LVEF: left ventricular ejection fraction.

ROC curve analysis identified an optimal EASIX cut-off value of >0.93 for predicting CIN, with the corresponding AUC indicating very good discriminatory performance (*p* < 0.001, AUC = 0.785, 95% CI 0.738–0.832). An EASIX cut-off value > 0.93 predicted CIN occurrence with 72% sensitivity and 72% specificity (Figure 1).

**Figure 2:**
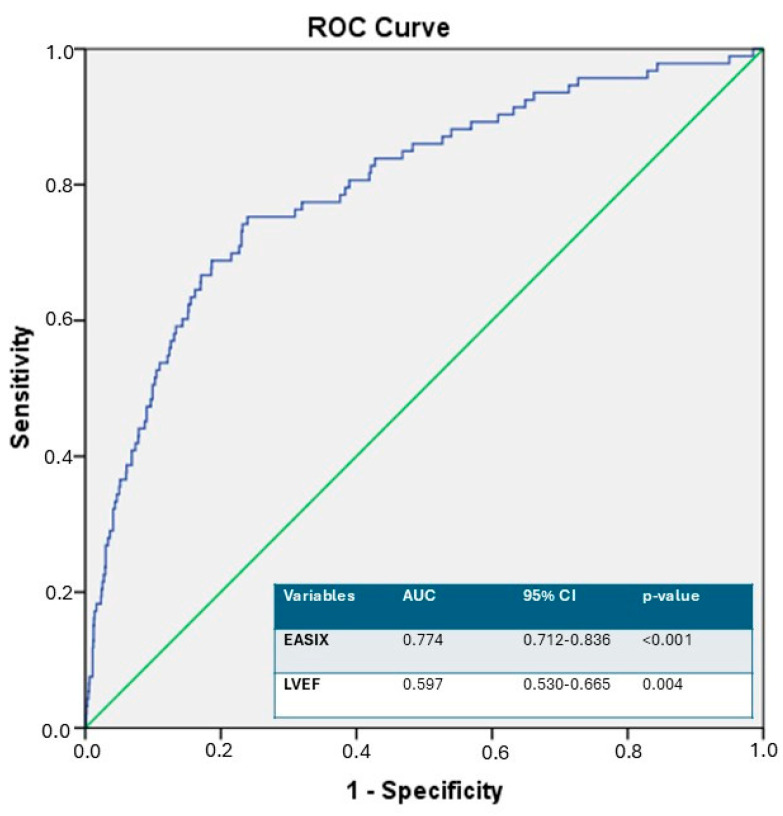
The ROC curve analysis of EASIX for predicting in-hospital mortality. ROC: receiver operating characteristic, EASIX: endothelial activation and stress index, LVEF: left ventricular ejection fraction.

**Figure 3:**
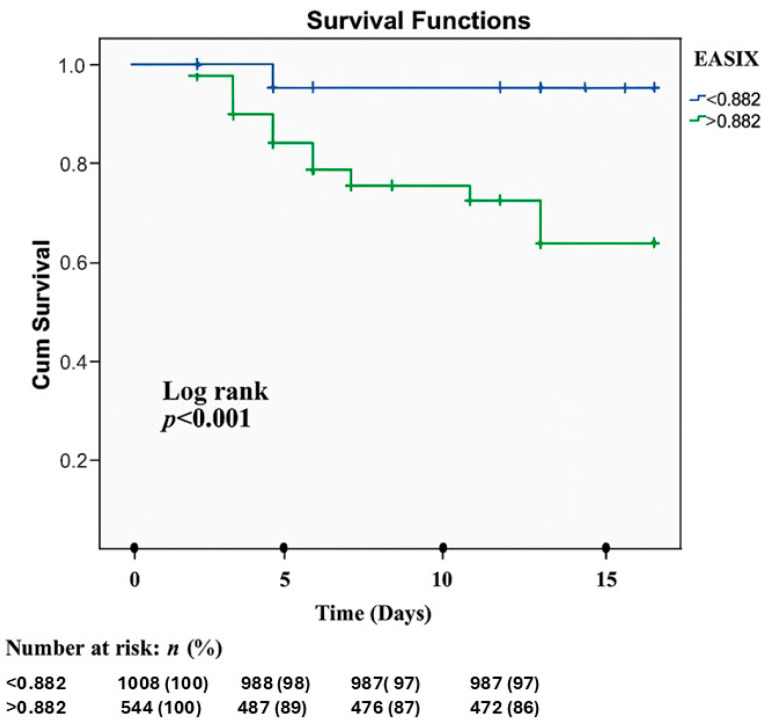
Kaplan–Meier survival analysis for in-hospital mortality according to the EASIX ROC-derived cut-off value. ROC: receiver operating characteristic, EASIX: endothelial activation and stress index.

**Figure 4:**
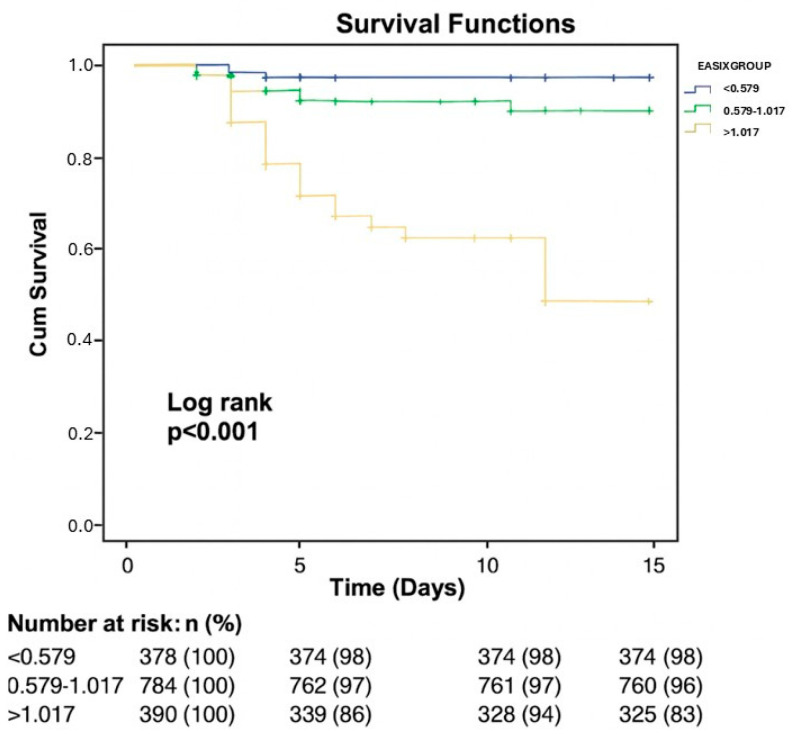
Kaplan–Meier survival analysis for in-hospital mortality according to the EASIX percentile-based cut-off value. EASIX: endothelial activation and stress index.

Univariable and multivariable logistic regression analyses for predictors of in-hospital mortality are presented in Table 5. Univariable analysis indicated that age (*p* < 0.001; OR 1.038 95% CI 1.019–1.057), diabetes mellitus (*p* = 0.002; OR 2.003 95% CI 1.290–3.109), failed PCI (*p* < 0.001; OR 3.315 95% CI 1.825–6.021), LVEF (*p* < 0.001; OR 0.963 95% CI 0.943–0.984), CIN (*p* < 0.001; OR 3.262 95% CI 1.876–5.671), and EASIX (*p* < 0.001; OR 1.711 95% CI 1.309–2.235) showed significant associations with in-hospital mortality. Multivariable analysis revealed that failed PCI (*p* = 0.005; OR 2.869, 95% CI 1.370–6.009), LVEF (*p* = 0.018; OR 0.974, 95% CI 0.953–0.996), and EASIX (*p* < 0.001; OR 3.592, 95% CI 2.503–5.157) emerged as independent predictors for in-hospital mortality. ROC curve analysis identified an optimal EASIX cut-off value of >0.88 for the prediction of in-hospital mortality, with the corresponding AUC demonstrating very good discriminatory performance. (*p* < 0.001, AUC = 0.774, 95% CI 0.712–0.836) (Figure 2). At the threshold, EASIX > 0.88 predicted in-hospital mortality with a sensitivity of 77% and specificity of 77%. Kaplan–Meier survival analysis demonstrated significant differences in in-hospital mortality between groups stratified according to the ROC-derived EASIX cut-off value (Figure 3 and Figure 4, respectively).

## 4. Discussion

In this retrospective study, we investigated the prognostic utility of the EASIX for predicting CIN and in-hospital mortality in patients with STEMI undergoing primary PCI. Our principal findings were as follows: higher EASIX values were strongly associated with CIN development; EASIX independently predicted CIN after accounting for traditional risk factors, including age, CKD, and reduced LVEF; and EASIX independently predicted in-hospital mortality with an accuracy superior to traditional clinical variables. These results highlight EASIX as a readily available and clinically meaningful biomarker that reflects endothelial activation and systemic stress in the AMI setting.

Although multiple risk factors contributing to the development of CIN were identified, its prediction remains challenging in the clinical setting due to significant interpatient variability in susceptibility to the nephrotoxic effects of contrast media [26]. CIN remains a common and clinically significant complication after primary PCI, particularly in STEMI, where hemodynamic instability, high thrombus burden, and the need for rapid reperfusion frequently limit the application of preventive strategies [27,28]. Consistent with previous literature, older age, CKD, and lower LVEF were independently associated with CIN in our cohort. The incidence of CIN (7.6%) fell within the range reported in other STEMI populations, supporting the representativeness of our sample.

Growing evidence highlights endothelial dysfunction and inflammation as key contributors to the pathogenesis of CIN [29]. Contrast exposure can trigger renal vasoconstriction, oxidative stress, and tubular epithelial injury, ultimately activating the endothelium [29,30]. Activated endothelial cells increase vascular permeability, upregulate adhesion molecules, and reduce nitric oxide bioavailability, collectively contributing to renal microcirculatory impairment [30]. EASIX, which incorporates serum LDH, serum creatinine, and platelet count, has emerged as a promising, readily accessible composite biomarker that integrates markers of cellular injury, renal impairment, and endothelial activation [31]. Accordingly, the strong association of EASIX with CIN observed in the present study appears to be biologically plausible. Although originally developed to predict mortality in hematologic malignancies and post-transplant settings, EASIX has recently emerged as a promising biomarker in many cardiovascular and critical care populations [14,15,16,18,19]. Subsequent studies have demonstrated that EASIX carries prognostic significance across a broad range of clinical conditions, including solid organ cancers, diabetes, rheumatologic and dermatologic diseases, sepsis, and asthma [32,33,34,35]. These data suggest that EASIX reflects a shared pathway of endothelial activation and systemic stress. Its clinical relevance has also been confirmed in cardiovascular and renal disorders.

In stable coronary artery disease, EASIX independently predicted mortality, while in AMI, it was strongly associated with 30-day mortality [36]. It also served as an independent predictor of long-term mortality in patients undergoing transcatheter aortic valve replacement and in those with chronic HF [37,38,39]. Surgical cohorts show similar findings: higher EASIX levels were linked to postoperative morbidity and poorer survival in patients undergoing coronary artery bypass grafting [40]. Moreover, analyses of the MIMIC-IV database revealed that greater EASIX values correlate with greater in-hospital and long-term mortality among critically ill patients, including those with atrial fibrillation [41,42]. EASIX has additionally been linked to stroke risk and overall mortality and was shown to predict AKI and short-term adverse outcomes in elderly critically ill patients [43]. Together, these findings highlight EASIX as a versatile and robust biomarker applicable across diverse clinical settings.

Importantly, EASIX showed a high discriminative ability for CIN (AUC 0.785) using a cut-off > 0.93, with balanced sensitivity and specificity (72% and 72%, respectively). This performance is notable given that currently available CIN risk scores are often cumbersome for use in emergent settings or are limited by the need for multiple clinical variables. EASIX, by contrast, can be calculated rapidly from routine laboratory parameters available upon admission, making it particularly practical in the acute STEMI workflow. Our study also revealed that EASIX independently predicted in-hospital mortality. This association may reflect the broader pathophysiological significance of endothelial dysfunction in STEMI, where microvascular dysfunction, reperfusion injury, systemic inflammation, and multi-organ stress collectively influence outcomes. The cut-off value of >0.88 showed strong discriminatory performance (AUC 0.774) and enabled clear separation of survival curves on Kaplan–Meier analysis. These findings align with previous studies reporting the EASIX’s prognostic relevance in AMI, TAVR patients, and critically ill cohorts, further supporting its utility across diverse cardiovascular settings [34,37,38,44].

The relationship between elevated EASIX and poor outcomes may also be partly explained by the interplay between endothelial dysfunction and the inflammatory response during myocardial ischemia–reperfusion. Elevated LDH levels reflect cellular injury, creatinine indicates impaired renal perfusion or pre-existing dysfunction, and platelet count contributes to thrombo-inflammatory pathways—each of which is strongly implicated in the progression of CIN, microvascular obstruction, and mortality after STEMI.

Strengths and clinical implications of our study include the large STEMI cohort, uniform use of a single low-osmolar contrast agent, and the real-world applicability of readily obtainable laboratory markers. EASIX could be incorporated into early risk stratification to identify high-risk patients who may benefit from aggressive hydration strategies, minimization of contrast volume, closer renal monitoring, and more intensive hemodynamic optimization. Its ease of calculation allows seamless integration into emergency and catheterization laboratory workflows, where time-sensitive decision-making is critical.

## 5. Study Limitations

This study has some limitations. First, the study’s retrospective, single-center nature could restrict the applicability of the results and lead to selection bias. Despite adjusting for major confounders, unmeasured variables, such as peri-procedural hemodynamic parameters, precise timing of medications, and operator-related differences, may have influenced the outcomes. Although the number of patients with cardiogenic shock and/or acute HF who did not receive periprocedural hydration was only a small proportion of the study population, this factor may still have influenced the study outcomes. Also, the study evaluated only in-hospital events; therefore, long-term renal outcomes and post-discharge mortality could not be assessed. Finally, although EASIX is a simple and practical index, its components may be affected by acute systemic conditions unrelated to endothelial dysfunction, which could impact its discriminatory performance. For future perspective, multicenter research is necessary to confirm these results and establish standardized cut-off values for clinical application.

## 6. Conclusions

The present study found that EASIX independently and strongly predicted CIN and in-hospital mortality in STEMI patients who underwent primary PCI. CIN is a clinically relevant angiographic complication known to prolong hospital stay and significantly increase short-term mortality; therefore, timely detection of high-risk patients is crucial. By integrating markers of endothelial activation, systemic stress, and renal function, EASIX provides prognostic information beyond conventional clinical parameters and can be calculated rapidly from routine laboratory tests [14,31].

Given its simplicity, availability, and strong discriminatory performance, EASIX could be useful for early risk stratification in the acute STEMI setting, guiding clinicians in identifying high-risk patients who could require more intensified preventive strategies, careful contrast management, and closer monitoring. Prospective multicenter trials are warranted to verify these outcomes and to establish standardized EASIX thresholds for widespread clinical use.

## Figures and Tables

**Table 1:** Baseline demographic, clinical, and laboratory characteristics of the patients with and without contrast-induced nephropathy.

Variables	CIN (−) (*n* = 1434)	CIN (+) (*n* = 118)	*p*-Value
Age, years	58.08 ± 11.46	64.85 ± 12.40	<0.001
Gender (male), *n* (%)	1114 (77.7)	86 (72.9)	0.231
Diabetes, *n* (%)	313 (21.8)	47 (39.8)	<0.001
Systolic blood pressure, mmHg	128.9 ± 27.2	118.3 ± 25.6	0.279
Diastolic blood pressure, mmHg	68.6 ± 21.8	62.1 ± 13.8	0.572
Hypertension, *n* (%)	425 (29.6)	28 (23.7)	0.175
Smoking, *n* (%)	541 (37.7)	45 (38.1)	0.930
COPD, *n* (%)	16 (1.1)	3 (2.5)	0.176
CKD, *n* (%)	15 (1.0)	10 (8.5)	<0.001
GPIIb/IIIa inh usage, *n* (%)	729 (50.8)	63 (53.4)	0.594
Chronic HF, *n* (%)	72 (5.0)	5 (4.2)	0.706
Prior PCI, *n* (%)	191 (13.3)	18 (15.3)	0.554
Contrast agent amount, mL	247.65 ± 85.77	252.11 ± 93.68	0.589
LVEF (%)	47.85 ± 9.94	42.95 ± 13.30	<0.001
Beta-blocker, *n* (%)	344 (24.3)	31 (27.4)	0.450
ACEi or ARB, *n* (%)	474 (33.4)	44 (38.9)	0.233
Statin, *n* (%)	560 (40.7)	51 (45.9)	0.282
Oral antidiabetics, n (%)	294 (20.7)	37 (31.4)	0.007
Hemodialysis, *n* (%)	0 (0)	12 (10.2)	<0.001
In-hospital death, *n* (%)	75 (5.2)	18(15.3)	<0.001
Cardiogenic shock and/or acute HF, *n* (%)	92 (6.4)	11 (9.3)	0.223
Culprit vessel, *n* (%)			0.526
-LMCA	55 (3.8)	7 (5.9)	
-LAD artery	464 (32.4)	39 (33.1%)	
-Cx artery	214 (14.9)	12 (10.2%)	
-RCA	674 (47.0)	57 (48.3)	
-Bypass graft	27 (1.9)	3 (2.5)	
Failed PCI, *n* (%)	82 (5.7)	13 (11)	0.021
Follow-up period, *days*	3.80 ± 2.28	5.79 ± 3.31	<0.001

Values are presented as mean ± standard deviation or number (%). ACEi: angiotensin-converting enzyme inhibitor, ARB: angiotensin receptor blocker, CKD: chronic kidney disease, COPD: chronic obstructive pulmonary disease, Cx: circumflex, HF: heart failure, LAD: left anterior descending, LMCA: left main coronary artery, LVEF: left ventricular ejection fraction, PCI: percutaneous coronary intervention.

**Table 2:** Laboratory results of the study population.

Variables	CIN (−) (*n* = 1434)	CIN (+) (*n* = 118)	*p*-Value
Peak Troponin I, ng/mL	3.68 ± 1.70	3.91 ± 1.68	0.092
WBC, 10^3^/mm^3^	11.40 (0.90–46.90)	11.50 (4.30–23.80)	0.920
HGB, g/dL	13.48 ± 1.76	12.42 ± 1.81	0.728
Platelets, 10^3^/mm^3^	234.78 ± 69.50	247.14 ± 81.50	0.006
Neutrophils, 10^3^/mm^3^	9.12 (0.45–30.70)	9.64 (2.61–40.90)	0.152
CRP, mg/L	7 (0–311)	7 (0.1–170)	0.776
Albumin, g/dL	3.56 ± 0.51	3.49 ± 0.58	0.129
Glucose, mg/dL	133 (53–693)	170 (90–608)	<0.001
BUN, mg/dL	16.85 ± 5.43	21.78 ± 7.23	<0.001
Creatinine, mg/dL	0.88 ± 0.27	1.20 ± 0.39	<0.001
GFR, mL/min/1.73 m^2^	99.16 ± 29.35	72.36 ± 23.77	<0.001
AST, U/L	63 (6–3429)	58 (11–490)	0.893
ALT, U/L	25 (0–580)	26 (0–3512)	0.070
LDH, U/L	200.51 ± 34.33	256.48 ± 41.24	<0.001
Triglycerides, mg/dL	131 (11–1312)	156 (47–500)	0.002
HDL, mg/dL	38.71 ± 10.33	37.42 ± 11.59	0.295
LDL, mg/dL	109.20 ± 36.39	122.10 ± 51.68	0.019
EASIX	0.74 (0.14–35.96)	1.24 (0.46–5.27)	<0.001

Values are presented as mean ± standard deviation or number (%). ALT: alanine aminotransferase, AST: aspartate aminotransferase, BUN: blood urea nitrogen, CRP: C-reactive protein, GFR: glomerular filtration rate, HDL: high-density lipoprotein, LDH: lactate dehydrogenase, LDL: low-density lipoprotein, WBC: white blood cell.

**Table 3:** Three-group stratification based on the 25th and 75th percentiles of the EASIX score.

Variables	<0.57 (<25th Percentile) *n* = 378	0.579–1.017 (25th–75th Percentile) *n* = 784	>1.017 (>75th Percentile) *n* = 390	*p*-Value
Age, years	56.53 ± 11.76 ^a^	58.04 ± 11.30 ^a^	61.71 ± 11.73 ^b^	<0.001
Gender (male), *n* (%)	243 (64.3) ^a^	624 (79.6) ^b^	333 (85.4) ^c^	<0.001
Diabetes	74 (19.6) ^a^	162 (20.7) ^a^	124 (31.8) ^b^	<0.001
Hypertension	112 (29.6)	240 (30.6)	101 (25.9)	0.241
Smoking	146 (38.6)	275 (35.1)	165 (42.3)	0.051
COPD	6 (1.6) ^a,b^	4 (0.5) ^b^	9 (2.3) ^a^	0.023
CKD	0 (0) ^a^	3 (0.4) ^a^	22 (5.6) ^b^	<0.001
GPIIb/IIIa inh usage	192 (50.8)	413 (52.7)	187 (47.9)	0.310
Chronic HF	16 (4.2)	34 (4.3)	27 (6.9)	0.119
Prior PCI	55 (14.6)	92 (11.7)	62 (15.9)	0.112
Contrast agent amount	243.38 ± 89.55	251.95 ± 88.06	244.48 ± 79.37	0.186
LVEF, %	47.71 ± 9.91 ^a,b^	48.17 ± 9.89 ^a^	45.80 ± 11.40 ^b^	0.003
Beta-blocker	100 (26.7)	186 (24)	89 (23.3)	0.511
ACEi or ARB	139 (37.1)	254 (32.8)	125 (32.7)	0.314
Statin	167 (46)	297 (39.4)	147 (39.7)	0.093
Oral antidiabetics	78 (20.9)	168 (21.6)	85 (22)	0.928
Peak Troponin I, ng/mL	3.56 ± 1.74	3.72 ± 1.69	3.78 ± 1.66	0.169
WBC, 10^3^/mm^3^	11.20 (0.90–46)	11.60 (3.90–32)	11.30 (3.33–32)	0.867
HGB, g/dL	13.35 ± 1.87	13.50 ± 1.72	13.55 ± 1.76	0.260
Platelets, 10^3^/mm^3^	296.54 ± 81.50 ^a^	230.88 ± 49.46 ^b^	186.65 ± 49.31 ^c^	<0.001
Neutrophils, 10^3^/mm^3^	9.6 (1.9–26) ^a^	9.3 (0.45–30) ^a^	8.68 (1.8–40) ^b^	<0.001
CRP, mg/L	7 (0–311)	7 (0–308)	7 (0–253)	0.570
Albumin, g/dL	3.53 ± 0.51	3.56 ± 0.54	3.58 ± 0.47	0.389
Glucose, mg/dL	133 (53–693)	170 (90–608)		0.004
BUN, mg/dL	14.91 ± 4.21 ^a^	16.55 ± 4.96 ^b^	20.84 ± 8.03 ^c^	<0.001
Creatinine, mg/dL	0.71 ± 0.14 ^a^	0.87 ± 0.18 ^b^	1.15 ± 0.41 ^c^	<0.001
GFR, mL/min/1.73 m^2^	117.00 ± 35.57 ^a^	97.53 ± 21.94 ^b^	77.08 ± 24.07 ^c^	<0.001
AST, U/L	62 (7–1174)	61 (6–3429)	65 (11–1085)	0.438
ALT, U/L	25 (0–234)	26 (0–472)	25 (0–3512)	0.407
LDH, U/L	182.74 ± 34.81 ^a^	203.81 ± 30.83 ^b^	228.04 ± 40.34 ^c^	<0.001
Triglycerides, mg/dL	130 (36–848)	136 (11–1312)	128 (36–574)	0.466
HDL, mg/dL	39.12 ± 11.28	38.35 ± 9.83	38.68 ± 10.76	0.544
LDL, mg/dL	108.02 ± 37.44	110.72 ± 37.05	110.86 ± 41.22	0.533
Hemodialysis	0 (0)	0 (0)	12 (3.1)	<0.001
CIN	6 (1.6)	33 (4.2)	79 (20.3)	<0.001
In-hospital death	4 (1.1)	24 (3.1)	65 (16.7)	<0.001
Failed PCI	19 (5)	46 (5.9)	30 (7.7)	0.279
Follow-up period, days	3.81 ± 2.26	3.97 ± 2.56	4.03 ± 2.33	0.431

Values are presented as mean ± standard deviation or number (%). ^a,b,c^ different superscript markers indicate clinical significance between groups. ACEi: angiotensin-converting enzyme inhibitor, ALT: alanine aminotransferase, ARB: angiotensin receptor blocker, AST: aspartate aminotransferase, BUN: blood urea nitrogen, CIN: contrast-induced nephropathy, CKD: chronic kidney disease, COPD: chronic obstructive pulmonary disease, CRP: C-reactive protein, HDL: high-density lipoprotein, GFR: glomerular filtration rate, HF: heart failure, HGB: hemoglobin, LVEF: left ventricular ejection fraction, LDH: lactate dehydrogenase, LDL: low-density lipoprotein, PCI: percutaneous coronary intervention, WBC: white blood cell.

**Table 4:** Logistic regression analysis for predicting contrast-induced nephropathy in the study patients.

	Univariate Analysis		Multivariate Analysis	
Variable	OR (95% CI)	*p*-Value	OR (95% CI)	*p*-Value
Age	1.052 (1.035–1.070)	<0.001	1.053 (1.031–1.075)	<0.001
DM	2.371 (1.606–3.499)	<0.001		
Failed PCI	2.041 (1.100–3.787)	0.024		
LVEF	0.958 (0.940–0.976)	<0.001	0.965 (0.944–0.986)	0.001
LDL	1.008 (1.003–1.013)	0.002	1.005 (0.999–1.012)	0.086
CKD	1.225 (1.089–1.361)	<0.001	1.338 (1.098–1.678)	0.045
EASIX	1.771 (1.370–2.290)	<0.001	2.467 (1.709–3.560)	<0.001

CI: confidence interval, CKD: chronic kidney disease, DM: diabetes mellitus, EASIX: endothelial activation and stress index, LVEF: left ventricular ejection fraction, LDL: low-density lipoprotein, OR: odds ratio, PCI: percutaneous coronary intervention.

**Table 5:** Logistic regression analyses for predictors of in-hospital mortality in the study patients.

	Univariate Analysis		Multivariate Analysis	
Variable	OR (95% CI)	*p*-Value	OR (95% CI)	*p*-Value
Age	1.038 (1.019–1.057)	<0.001	1.020 (0.999–1.042)	0.064
DM	2.003 (1.290–3.109)	0.002	1.634 (0.974–2.741)	0.063
Failed PCI	3.315 (1.825–6.021)	<0.001	2.869 (1.370–6.009)	0.005
LVEF	0.963 (0.943–0.984)	<0.001	0.974 (0.953–0.996)	0.018
LDL	0.995 (0.988–1.001)	0.126		
CIN	3.262 (1.876–5.671)	<0.001		
EASIX	1.711 (1.309–2.235)	<0.001	3.592 (2.503–5.157)	<0.001

CI: confidence interval, CIN: contrast-induced nephropathy, DM: diabetes mellitus, EASIX: endothelial activation and stress index, LVEF: left ventricular ejection fraction, LDL: low-density lipoprotein, OR: odds ratio, PCI: percutaneous coronary intervention.

## Data Availability

The data presented in this study are available from the corresponding author upon reasonable request.
